# Measuring resilience to chronic pain in population surveys using hair cortisol

**DOI:** 10.1017/S0033291725101049

**Published:** 2025-08-15

**Authors:** Tarani Chandola, Stephanie Cahill, Wanying Ling, Meena Kumari

**Affiliations:** 1 Department of Sociology, The University of Hong Kong, Hong Kong, China; 2 Manchester Institute of Education, University of Manchester, Manchester, UK; 3 Institute for Social and Economic Research, University of Essex, Colchester, UK

**Keywords:** chronic pain, cortisol, mental health, resilience, stress

## Abstract

**Background:**

Chronic pain activates the HPA axis stress response resulting in the release of cortisol, although empirical associations are often contradictory. Quantile regression models of hair cortisol may help us measure HPA-axis dysregulation more accurately and establish more robust associations with chronic pain. We also examined whether people with chronic pain characterised by HPA-axis dysregulation are at risk of future mental ill-health.

**Methods:**

This study examined data from the English Longitudinal Study of Ageing (ELSA, *n* = 4,560) and the UK Household Longitudinal Survey-Innovation Panel (UKHLS-IP, *n* = 473) to assess whether quantile regression methods enable us to assess more robust associations between hair cortisol and chronic pain, and whether older adults with chronic pain characterised by HPA-axis dysregulation are at risk of future mental ill-health.

**Results:**

In ELSA, chronic pain was associated with a 15% (CI: 6%–23%) increase in cortisol at the 10th percentile of the hair cortisol distribution among older adults and a 19% (CI: 2%–37%) increase at the 80th percentile, but no association was found at the 30th or 40th percentiles. Having a low cortisol response to chronic pain protected against the recurrence of depression. These patterns of association were replicated in the UKHLS-IP sample.

**Conclusions:**

The associations demonstrated across two longitudinal population surveys from the UK indicate that quantile regression analysis of hair cortisol may be useful in identifying individuals resilient to chronic pain. Hair cortisol is a promising biomarker that can be measured in population studies to quantify the stress response and resilience to future mental ill-health.

## Introduction

Pain is a powerful stressor that activates the body’s major stress response system, the hypothalamic–pituitary–adrenal (HPA) axis, to release the stress hormone cortisol (Ulrich-Lai & Herman, 2009). When stress is prolonged (chronic), the HPA axis can become dysregulated, resulting in either a hypo- or hyperactive system (Miller, Chen, & Zhou, 2007). However, empirical data on the dysregulation of the HPA axis and cortisol levels in chronic pain do not fit any consistent patterns (Abdallah & Geha, 2017). Some studies of chronic pain conditions reported increases in salivary cortisol among people with chronic back pain (Sveinsdottir et al., 2016; Vachon-Presseau, Roy et al., 2013; Vachon-Presseau, Martel et al., 2013) and neck, shoulder, and back pain (Schell et al., 2008), and increased plasma cortisol levels among patients with chronic migraine pain (Rainero et al., 2006). Other studies have reported decreases in salivary cortisol levels among people with chronic back pain (Muhtz et al., 2013), chronic neck pain (Shahidi, Sannes, Laudenslager, & Maluf, 2015), fibromyalgia and chronic musculoskeletal pain (Generaal et al., 2014; Riva et al., 2010), chronic pelvic pain syndrome (Lundh et al., 2013), and decreased plasma cortisol levels, among women with complex regional pain syndrome (Buryanov, Kostrub, & Kotiuk, 2017). Furthermore, additional studies have reported no associations between salivary cortisol and fibromyalgia (Nes et al., 2010) and chronic whiplash-associated disorders (Meeus et al., 2015). Similarly, no associations between plasma cortisol levels and chronic pelvic pain were reported (Wingenfeld et al., 2009). Moreover, there was conflicting evidence for a relationship between cortisol and pain in a systematic review of people with osteoarthritis (Villafañe et al., 2020). Nearly all the studies have relatively small sample sizes (around 100 or fewer), which reduces statistical power and makes it hard to generalize to the wider population.

**Figure 1.**
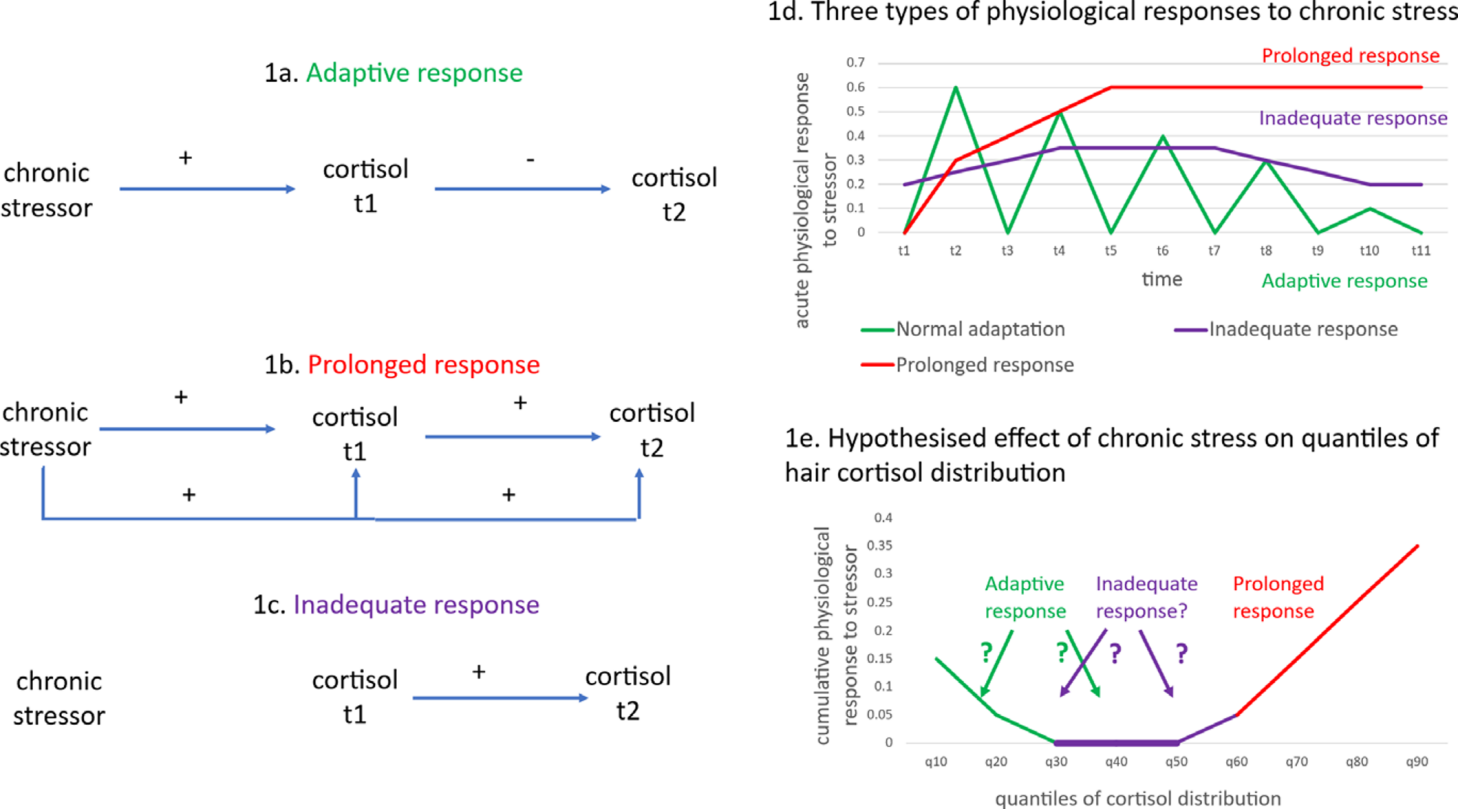
Differential effects of a chronic stressor on cortisol. Figure 1 (adapted from McEwen (2000)) represents the three patterns of cortisol responses to stressors (adaptive, prolonged, and inadequate responses) that correspond to the different stages of allostasis and allostatic load. In the case of chronic stress, where the stressor persists but fluctuates, an adaptive response (Figure 1a) requires some cortisol production initially, but this reduces after time – see the green line in Figure 1d. Cortisol is produced through activation of the HPA axis after exposure to a stressor (see the positive effect of the chronic stressor on cortisol at t1 in Figure 1a). However, the negative feedback loop in the HPA axis results in cortisol production being reduced when the levels of cortisol are too high (see the negative effect of cortisol at t1 on cortisol at t2 in Figure 1a). The initial positive effect on cortisol levels and the negative feedback loop on further cortisol release are adaptive responses that maintain allostasis. This adaptive response indicates resilience, marked by physiologically appropriate, moderated cortisol secretion. Hyperarousal refers to an exaggerated (or ‘prolonged’) stress response. The prolonged response is marked by a sustained production of stress hormones that does not rapidly decline post-exposure to the stressor – see Figure 1b and the red line in Figure 1d – which may indicate a lack of internal resources to cope with chronic stress, and the continued release of cortisol as a result of the chronic stressor (see the continuing positive effects of chronic stress on cortisol at t1 and t2 in Figure 1b). Hypo-arousal or an inadequate response is a stage where extreme exposure to stressors results in insufficient stress hormone production – see Figure 1c and the purple line in Figure 1d. The inadequate response suggests dysregulation or failure to mount an effective physiological response to the chronic stressor – see the lack of causal arrows from chronic stressor to cortisol at t1 or t2 in Figure 1c.

One explanation for the conflicting reports could be the difficulty in distinguishing between different patterns of adaptive and dysregulated cortisol responses to stressors. The HPA axis is normally controlled by a negative feedback system in which circulating cortisol inhibits the release of more cortisol after an initial increase in cortisol following exposure to a stressor (Figure 1a – the adaptive response). HPA axis dysregulation is characterized by either excessive (Figure 1b – the prolonged response) or a lack of cortisol production (Figure 1c – the inadequate response), resulting in an allostatic load (Figure 1d).

The allostatic load model is based on how our bodies respond (in terms of physiological responses) to repeated exposures to stressors over time. Most of the chronic pain studies reviewed above measured salivary cortisol. However, as there is a large diurnal variation in the HPA axis, time-specific measures, such as salivary cortisol, may reflect diurnal influences rather than the effect of chronic stressors. Many existing studies have not measured waking cortisol responses or how HPA axis production changes over the day (Villafañe et al., 2020). Cortisol measured from hair, on the other hand, measures HPA axis hormone production over multiple weeks or months, enabling an integrated assessment of long-term cortisol production (Kirschbaum, Tietze, Skoluda, & Dettenborn, 2009). This reduces methodological issues related to diurnal variability; however, with an integrated (or cumulative) measure of cortisol, it is not easy to infer whether a low value of hair cortisol corresponds to an inadequate or an adaptive response. In the case of chronic stress, where the stressor persists but fluctuates, an adaptive response requires some cortisol production, but this reduces over time. In contrast, an ‘inadequate response’ would reflect insufficient cortisol production, even as the stressor persists but fluctuates over time. Therefore, in chronic stress scenarios, an adaptive response indicates resilience, marked by physiologically appropriate, moderated cortisol secretion, whereas inadequate responses suggest dysregulation or failure to mount an effective physiological response. This study attempts to distinguish between these two responses when interpreting low values of hair cortisol.

Measuring dysregulation in cortisol production necessitates the use of statistical methods that go beyond a focus on average levels of cortisol. A focus on effects at the mean can result in important effects being ignored among people at the extremes of the cortisol distribution, even though it is at the extremes where we expect to find dysregulation of the HPA axis functioning. As an example, higher levels of neuroticism among middle-aged adults were associated with increased allostatic load, with the associations increasing in magnitude among adults at the high end of the distribution of physiological ‘wear and tear’ (Atkins, Turner, Chandola, & Sutton, 2020).

We use quantile regression methods, specifically the Recentered Influence Function (RIF) regression approach (Firpo, Fortin, & Lemieux, 2009), to assess the relationship between cortisol levels and chronic pain among adults at different points of the hair cortisol distribution. Standard regression models estimate the effect of an explanatory variable, *X*, on the population unconditional mean of an outcome variable, *Y.* Quantile regression methods enable estimation at specific percentiles (or ‘quantiles’) of the distribution of *Y.* In relation to hair cortisol, quantile regression models may be particularly useful, as the effect of a stressor (such as chronic pain) may differ between adults with high and low levels of cortisol (or in other words, across different parts of the distribution of cortisol). For example, in any random sample of people, those with chronic pain, who have a prolonged response to cortisol, may be expected to be located at the higher ends of the hair cortisol distribution (the red line in Figure 1e). On the other hand, it is unclear whether people with an adaptive or inadequate response to chronic pain would result in patterns such that (a) the adaptive response is located at the lower end of the hair cortisol distribution and the inadequate response around the median, or (b) the inadequate response is located at the lower end of the hair cortisol distribution and the adaptive response at the median. It is also unclear whether the associations between chronic pain and cortisol would differ across the distribution of hair cortisol or whether a standard linear regression model of hair cortisol could adequately represent the association between chronic pain and hair cortisol.

***H1:*** Chronic pain is associated with hair cortisol, but the size of the association varies between people with low, middle, and high levels of hair cortisol.

If there is evidence for H1, it would be important to examine whether the differential associations correspond to HPA axis dysregulation and predicted full ill-health. An adaptive response to chronic pain (in terms of cortisol production) should correspond to a process of resilience that reduces the risk of future ill-health despite the experience of chronic pain. Although the constructs of positive adaptation and resilience are often used interchangeably, they are distinct concepts. Positive adaptation refers to the process of adjusting to new or stressful conditions in a way that minimizes harm and maintains or improves one’s level of functioning (Sturgeon & Zautra, 2013). Resilience, on the other hand, is a dynamic process that involves positive adaptation within the context of adversity, but also includes the capacity to recover quickly from difficulties, or ‘bounce back’ from adversity (Luthar & Cicchetti, 2000; Luthar, Cicchetti, & Becker, 2000). Viewing resilience as a trajectory of positive adaptation after a disturbance suggests that resilience is not just about the immediate response to adversity but also about the longer-term process of recovery and growth (Bonanno, 2005; Cahill, Hager, & Shryane, 2024; Galatzer-Levy, Huang, & Bonanno, 2018). The identification of biomarkers of resilience requires an evaluation of their predictive power in terms of future mental health (Walker et al., 2017). People with an adaptive response to chronic pain should be more likely to recover (or ‘bounce back’) from poor mental ill-health compared to people with an inadequate or prolonged response to chronic pain. An adaptive response would also include lower chances of incident mental ill-health, although given the strong association between chronic pain and mental ill-health, resilience is more likely among the recovery group rather than the incidence group.

***H2:*** Hair cortisol levels predict future poor mental ill-health among adults with chronic pain. Hair cortisol levels that correspond to lower levels of poor mental ill-health among adults with chronic pain indicate an adaptive response to chronic pain.

## Materials and methods

### Datasets

The data for this study came from the English Longitudinal Study of Ageing (ELSA) and the UK Household Longitudinal Survey-Innovation Panel (UKHLS-IP) surveys. ELSA is a panel study of individuals aged 50 years and older in England, which was initiated in 2002 with biennial follow-ups and a broadly representative sample. In wave 6 (2012–2013), hair samples for cortisol measurement were collected. The UKHLS-IP survey includes 1,500 households as a testbed for innovative data collection, similar to the main Understanding Society survey. In wave 12 (2019), 1,408 households were interviewed, yielding 2,162 participants who provided hair samples.

We conducted separate analyses in each dataset to compare the results. ELSA wave 6 data were used for cross-sectional cortisol analysis, and wave 9 data were used for follow-up on mental health changes. UKHLS-IP wave 12 data were used for cross-sectional cortisol analysis, and wave 13 data were used for follow-up on mental health. This dual approach offered both a snapshot and a longitudinal view of the relationship between cortisol levels and mental health outcomes. Detailed descriptions of the datasets and hair sample collection processes can be found in the SupplementaryAppendix.

### Variables

***Chronic pain.*** In the ELSA study, respondents were asked if they were often troubled with pain and its severity. A binary chronic pain variable was coded as 1 if the pain was moderate or severe most of the time. In addition, 96% of those with moderate or severe pain reported it lasting 3 months or longer. In the UKHLS-IP study, the Short-Form-12 (SF-12) pain question, ‘In the last 4 weeks, to what extent has pain interfered with your work?’ was used. Responses of ‘quite a bit’ to ‘extremely’ were coded as 1 in a binary pain variable, including those not in paid work (Patel et al., 2012).

***Mental ill-health.*** For the ELSA study, we used the Center for Epidemiologic Studies Depression scale (CES-D 8) questionnaire. According to previous research (Briggs et al., 2018), the cutoff point for depressive symptoms was established between scores of 0–3 and 4 or more. For UKHLS-IP, we used the General Health Questionnaire (GHQ-12) questionnaire, setting a cutoff point for poor mental health between scores of 0–2 and 3 or more (Goldberg et al., 1997).

***Hair cortisol.*** In the ELSA study, around 25% of the participants at wave 6, who were eligible for the hair sample collection, did not contribute, mainly because they had <2 cm of hair. In the UKHLS-IP study, the missingness of hair data was around 45%. For ELSA and UKHLS-IP, we initially performed a log transformation on the cortisol data to approximate a normal distribution. For the analysis predicting future mental ill-health, we transformed the log cortisol values into *z*-scores.

***Covariates.*** We considered a range of demographic and health-related variables to control for potential confounding and measurement error factors. In the ELSA study, the potential confounding variables included age, sex, education level, ethnicity, relationship status, moderate physical activity, adverse childhood experiences (such as the death of the mother when aged < 18 years and lived apart from both natural parents as a child), smoking status, and the number of medications taken, which could be common causes of chronic pain as well as cortisol levels. The measurement error factors include hair color, whether the hair had been dyed or chemically treated, season, and phase of cortisol collection, all of which have previously been shown to affect estimated levels of hair cortisol (Abell et al., 2016). Due to a smaller sample size in the UKHLS-IP study, our analysis was confined to a more limited set of variables, such as age, gender, and hair treatment. Descriptive statistics for these variables are presented in Table 1. The survey questions can be found in Table S5 in the Supplementary Appendix.

**Table 1.** Weighted descriptive statistics for the analytical samples from the ELSA and UKHLS-IP analyses

ELSA		UKHLS-IP	
Cortisol (mean, range)	24.99 [0.03,658.80]	Cortisol (mean, range)	59.96 [0.85,6871.50]
(log) cortisol (mean, range)	2.01 [−3.51,6.49]	(log) cortisol (mean, range)	2.21 [−0.16,8.84]
CESD–8 at w6 (%)		GHQ–12 caseness scores at w12 (%)	
CESD–8 score < 4	85.36	GHQ–12 caseness <3	83.63
CESD–8 score ≥ 4	14.38	GHQ–12 caseness ≥ 3	16.08
Missing	0.27	Missing	0.3
CESD–8 at w9 (%)		GHQ-caseness scores at w13 (%)	
CESD–8 score < 4	59.62	GHQ–12 caseness <3	59.54
CESD–8 score ≥ 4	9.5	GHQ–12 caseness ≥ 3	31.18
Missing	30.88	Missing	9.28
Chronic pain (%)		Pain (%)	
No severe	69.9	No severe	88.74
Moderate/severe pain	30.1	Quite a bit/extreme interference	11.26
Sex (%)		Sex (%)	
Male	33.11	Male	42.65
Female	66.89	Female	57.35
Age (mean, range)	65.96 [50,99]	Age (mean, range)	64.47 [50,93]
Whether dye or chemically treated hair (%)		Hair treated (%)	
Yes	41.46	No	62.88
No	58.54	Yes	37.12
Education level (%)			
Higher education and above	16.2		
Higher education below	46.25		
Foreign or others	11.08		
No qualification	26.46		
Relationship status (%)			
Married	64.03		
Cohabit	5.45		
Other	30.52		
Ethnicity (%)			
White	96.8		
Non-White	3.2		
Smoking status (%)			
Never smoked	39.89		
Ever smoked	46.89		
Current smoker	13.22		
Number of medications (%)			
None	26.89		
Once	13.2		
Twice	12.37		
Three times	9.7		
Four times or more	37.85		
Hair color (%)			
Gray	40.47		
Brown	26.04		
Blonde	14.76		
Other	18.72		
Phase (%)			
Phase 1	48.09		
Phase 2	51.91		
Season (%)			
Winter	26.5		
Spring	6.28		
Summer	24.91		
Fall	42.31		
Frequency of moderate activities (%)			
More than once a week	61.21		
Once a week	12.81		
One to three times a month	6.33		
Hardly ever or never	19.65		
Mother deceased when aged under 18 years (%)			
Alive	91.65		
Deceased	8.35		
Lived with both parents as a child (%)			
Lived with both parents	86.89		
Did not live with both parents	13.11		
*N* (observations)	4,539	*N* (observations)	473

*Note*: For the ELSA study, cortisol is measured in picograms per milliliter. For the UKHLS-IP study, cortisol is measured in picograms per milligram. Proportions are reported for categorical variables. Mean values are reported for continuous variables. Ranges (minimum  and maximum) are given in brackets.

### Analytical approaches

***Quantile regression models.*** We used the RIF method (Firpo et al., 2009) to estimate the association between chronic pain and hair cortisol among older adults at different quantiles of the unconditional (or the overall, uncontrolled) hair cortisol distribution (at the 10th, 20th, 30th, 40th, 50th, 60th, 70th, 80th, and 90th quantiles). Chronic pain was converted to *z*-statistics before analysis, such that the coefficient represents associations between cortisol and 1 standard deviation increase in chronic pain. The hair cortisol values were log transformed in order to achieve a more normal distribution. As small changes in the natural log of a variable are directly interpretable as percentage changes, to a very close approximation, using the log-transformed cortisol as a dependent variable in a regression model means that the estimated coefficients can be interpreted as a percentage change in cortisol.

***Logistic regression models.*** We used logistic regression models to predict cases of mental ill-health among older adults with chronic pain/pain interference, estimating the interaction effect between baseline mental ill-health and *z*-scores of the log cortisol values. The interaction term examined to what extent older adults with chronic pain differed in terms of future mental ill-health (incidence and recurrence) by levels of cortisol.

***Nonrandom attrition bias.*** Due to substantial attrition between waves 6 and 9 of the ELSA study, we used inverse probability weighting (IPW) to correct for nonrandom attrition bias under the missing at random assumption (Bhaskaran & Smeeth, 2014; Seaman & White, 2013). IPW re-weights the sample so that the characteristics of those who remain are, on average, similar to those of the full sample, making it a representative of both lost and retained individuals (Seaman & White, 2013).

***Longitudinal weights.*** We generated longitudinal weights (using IPW methods) starting with a response variable indicating missingness of mental ill-health data in follow-up waves (ELSA wave 9 and UKHLS-IP wave 13). Propensity scores for this response variable were then calculated using logistic regression, with the response variable as the dependent variable and a set of sociodemographic characteristics at baseline (ELSA wave 6 and UKHLS-IP wave 12) as independent variables. These propensity scores were then merged with the baseline cross-sectional analysis weights to produce follow-up weights that account for response (missingness). These weights were pruned and normalized to a sum of 1. A detailed description of the analytical approaches can be found in the Supplementary Appendix.

## Results

Figures S1 and S2 (Supplementary Appendix) are flowcharts describing how the analytical samples for the ELSA and UKHLS-IP analyses were derived. The characteristics of the analytical sample from wave 6 of the ELSA study are shown in Table 1. Around 30% of the sample reported moderate or severe pain most of the time, with nearly all of them (over 96%) reporting chronic pain lasting for more than 3 months. The ELSA sample had a mean age of 66 years, with a higher proportion of women. Over a quarter of the sample reported no educational qualifications, in line with the educational experiences of this cohort. There were very few people of non-White ethnicities. In all, 13% of the ELSA sample were current smokers. The mean number of medications reported was over 2. Nearly 42% of the sample reported having undergone some chemical treatment of their hair.

Table 1 also shows the characteristics of the analytical sample from the UKHLS-IP study. As the UKHLS-IP original sample includes children and young adults, the analytical sample was restricted to those aged 50 years and over. Nearly 11% of the UKHLS-IP participants reported pain that interfered with their work (‘quite a bit’ or ‘extremely’), and these included participants not in paid work.

**Figure 2.**
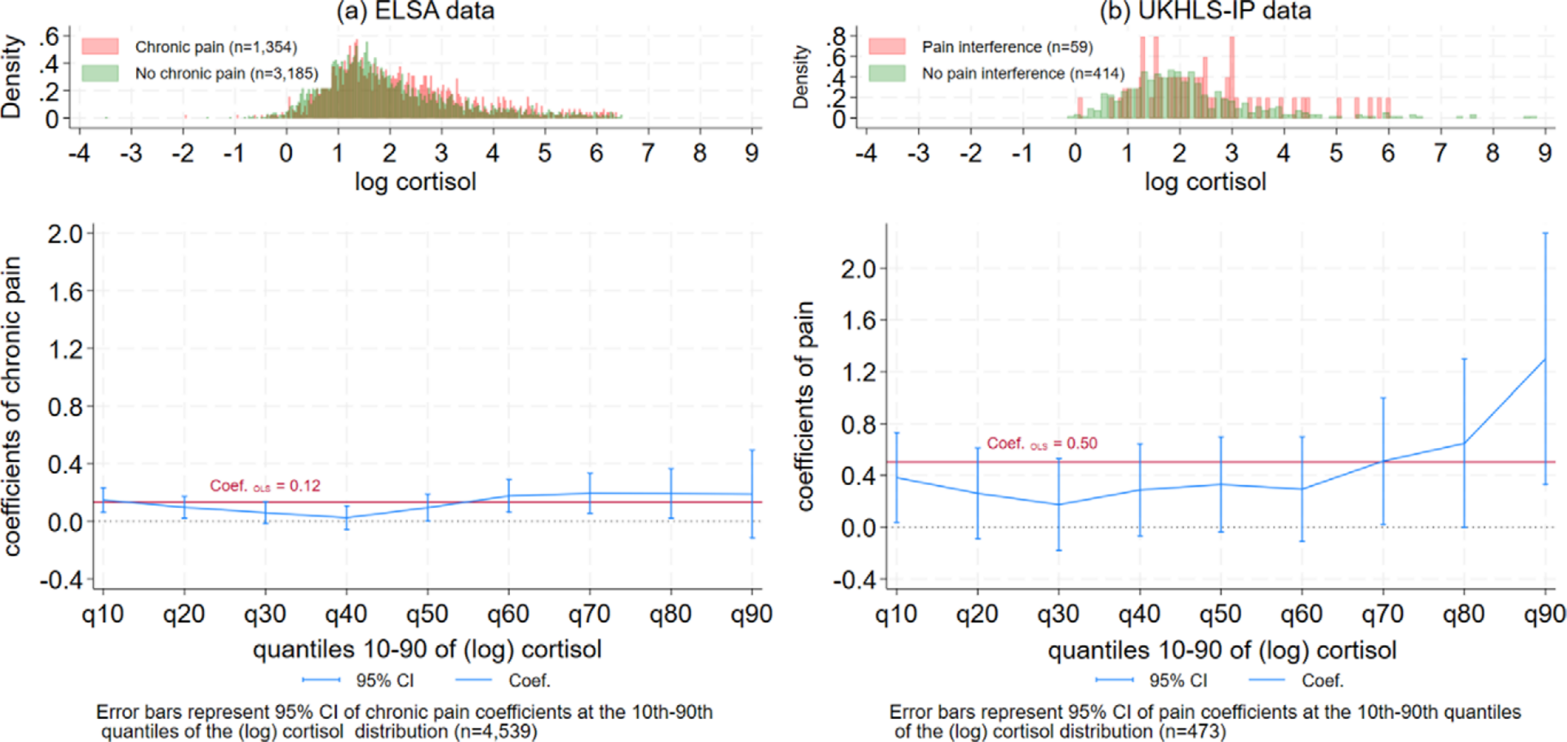
Distribution of (log) cortisol by pain and coefficients of the quantile regression model of (log) cortisol regressed on pain: Data from ELSA wave 6 (Figure 2a) and UKHLS-IP wave 12 (Figure 2b).

The results of the quantile regression models for the ELSA sample are shown in Table 2 (coefficients of chronic pain only) and Table S1 (Supplementary Appendix, with the full model coefficients). The estimate of chronic pain among older adults at the 10th quantile of the (log) cortisol distribution was 0.15 (or a 15% increase in the levels of cortisol for those with chronic pain), and it was between 0.10 and 0.19 at the 50th–90th quantiles. However, between the 30th and 40th quantiles of log cortisol, the estimate of chronic pain was close to zero. Figure 2a visualizes this U-shape association between the estimates of chronic pain coefficients by the (log) cortisol quantiles in the ELSA data – the estimates of pain were largest at the lowest and highest levels of the hair cortisol distribution, with no significant association between pain and cortisol observed at the 30th–40th quantiles of the cortisol distribution. At the 90th quantile of the cortisol distribution, the confidence intervals (CIs) were too wide for interpretation. The U-shape association is mirrored by the difference in the histograms of cortisol for people with chronic pain (in red) compared to those without chronic pain (in green). A higher proportion of older adults with chronic pain are located at the higher levels of the cortisol distribution, as well as some overrepresentation of those with chronic pain at very low levels of the cortisol distribution. Sex-specific analyses (Supplementary Figure S3) suggest similar patterns for men and women.

**Table 2.** Quantile regression coefficients of pain and (log) cortisol quantiles: ELSA w6 and UKHLS-IP

	ELSA		UKHLS-IP	
	Coef.	CI	Coef.	CI
Chronic pain/pain (Ref. = No severe)				
Quantile 10	0.15***	[0.06,0.23]	0.38*	[0.04,0.73]
Quantile 20	0.10*	[0.02,0.17]	0.26	[−0.09,0.61]
Quantile 30	0.06	[−0.02,0.14]	0.17	[−0.18,0.53]
Quantile 40	0.03	[−0.06,0.11]	0.29	[−0.07,0.64]
Quantile 50	0.10*	[0.00,0.19]	0.33	[−0.04,0.70]
Quantile 60	0.18**	[0.06,0.29]	0.29	[−0.11,0.70]
Quantile 70	0.20**	[0.06,0.33]	0.51*	[0.02,1.00]
Quantile 80	0.19*	[0.02,0.37]	0.65	[−0.00,1.30]
Quantile 90	0.19	[−0.11,0.49]	1.30**	[0.33,2.27]
*N* (observations)	4,539		473	

*Note*: For the ELSA study, chronic pain refers to moderate/severe interference, while for the UKHLS-IP study, pain refers to quite a bit/extreme interference. The ELSA analysis controlled for age, sex, education, ethnicity, relationship status, moderate physical activity, adverse childhood experiences (such as parental loss or separation before age 18 years), smoking status, the number of medications, and measurement error factors (such as hair color, hair treatment, season, and cortisol collection phase). For the UKHLS-IP study, the analysis was limited to age, gender, and hair treatment due to a smaller sample size. The 95% confidence intervals are given in brackets. *p  <  0.05, **p  <  0.01, ***p  <  0.001.

The estimates of the association between chronic pain and (log) cortisol derived through OLS regression models (the red line in Figure 2) are significantly higher than the quantile regression model estimates at the 30th–40th quantiles. This suggests standard linear regression models may not be appropriate for describing the heterogeneity in cortisol responses to chronic pain. All the regression models controlled for hair treatment and color, season, cortisol collection phase, age, sex, ethnicity, education qualifications, cohabitation with partner, smoking status, and the number of medications.

The results of the quantile regression models for the UKHLS-IP sample are shown in Table 2 (coefficients of pain level only) and Table S2 (Supplementary Appendix), with the full model coefficients). Pain interference was associated with a 38% increase in cortisol among older adults at the 10th quantile of the cortisol distribution, as well as a 51–130% increase in cortisol between the 70th and 90th quantiles. However, between the 20th and 60th quantiles, there was no statistically significant association between pain interference and (log) cortisol. Figure 2b visualizes the U-shape pattern of the quantile regression coefficients of pain by (log) cortisol quantiles in the UKHLS-IP wave 12 data. The patterns were similar to those shown in Figure 2a, although the estimated coefficients in the UKHLS-IP sample were larger compared to the ELSA sample, mainly because the latter included more covariates in the analysis that accounted for potential confounders of the effect of chronic pain on cortisol. Similar to the histograms in Figure 2a, older adults in the UKHLS-IP sample with pain interference were overrepresented at both the higher and the very low levels of the cortisol distribution.

The U-shape pattern in Figures 2a and 2b suggest that adults with a low cortisol response (at the 10th quantile) to chronic pain may have an adaptive response. Chronic pain appears to positively influence the levels of cortisol for this group of adults, although the levels of cortisol produced in response to their chronic pain are limited to the very low end of the cortisol distribution. Adults with a high cortisol response to pain (between the 60th and 90th quantiles) appear to have a prolonged cortisol response to chronic pain; chronic pain appears to result in elevated levels of cortisol at the highest ends of the cortisol distribution. For adults with moderate levels of cortisol (around the 30th – 40th quantiles), the association of chronic pain with cortisol production was close to zero. This fits the pattern of people with an inadequate response to chronic pain.

**Figure 3.**
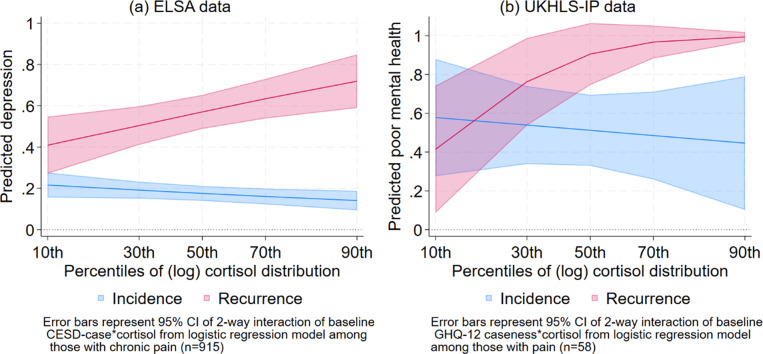
Predicted poor mental health (recurrence and incidence) by (log) cortisol percentiles among older adults with pain: Data from the ELSA (Figure 3a) and UKHLS-IP (Figure 3b) studies.

Table S3 (Supplementary Appendix) shows the results of the logistic regression model predicting risk of depression (from CES-D depressive symptoms) at ELSA wave 9 among older adults with chronic pain at baseline (wave 6), controlling for the interaction between baseline mental ill-health and *z*-scores of the log cortisol values, and other potential confounders. The interaction term was significantly different from zero (*p* < 0.01 in Supplementary Table S1), suggesting that the incidence and recurrence of depressive symptoms differed by baseline cortisol levels. Figure 3a visualizes the estimated proportion of CES-D depression cases at ELSA wave 9 by levels of cortisol, among people with and without depression at wave 6, as predicted by the model in Supplementary Table S3. The figure distinguishes between those **without** depression (the blue line) and those **with** depression (the red line) at baseline (wave 6). Unsurprisingly, older adults with depression at baseline are more likely to have higher rates of future depression than those without depression. The **recurrence** of depression (the red line) increased from lower to higher levels of cortisol. The predicted proportion of older adults with recurring depressive symptoms was around 40% (95% CI: 25–54%) at the 10th percentile of the cortisol distribution. At the 30th percentile of the cortisol distribution, the predicted proportion of recurring depressive symptoms was higher at 49% (95% CI: 39–58%), and the recurrence rate increased from 55% at the median to 71% at the 90th percentile of the cortisol distribution. On the other hand, the incidence of depression (the blue line) did not significantly vary across the cortisol distribution. Thus, there is clear evidence of lower rates of recurrence of depressive symptoms among older adults with chronic pain at the lowest levels of the cortisol distribution, compared to their peers with median to higher levels of cortisol.

Table S4 (Supplementary Appendix) shows the results of the logistic regression model predicting the risk of mental ill-health (from the GHQ-12) at UKHLS-IP wave 13 among older adults with pain interference, controlling for the interaction between baseline mental ill-health (at wave 6) and cortisol levels, as well as age and sex. Similar to Supplementary Table S3, the interaction term was significantly different from zero (*p* < 0.05). Figure 3b visualizes the estimated proportion of recurrent and **incident** mental ill-health at wave 13 by cortisol levels. These figures look very similar to their corresponding Figures from the ELSA analyses (Figure 3a). Figure 3b shows an increasing risk of recurrence of mental ill-health among older adults with pain interference (the red line) as cortisol levels increased from the 10th to the 90th percentiles of the cortisol distribution. This suggests that low levels of cortisol (in the first decile of the cortisol distribution) appear to enable older adults with pain to have greater chances of recovery (lower rates of recurrence) from mental ill-health, compared to their peers with higher levels of cortisol.

## Discussion

We found evidence for both our hypotheses. From the quantile regression models of cortisol, we established that the association of chronic pain and hair cortisol differed between older adults with low, middle, and high levels of hair cortisol (H1). The U-shape pattern of the coefficients of chronic pain across the distribution of hair cortisol suggests that older adults differ in their cortisol responses to chronic pain. Moreover, these differential cortisol responses to chronic pain were associated with future risks of recurring mental ill-health, with a low cortisol response to chronic pain predicting a greater recovery rate from mental ill-health. Low levels of cortisol produced in relation to chronic pain thus appear to be an adaptive response to chronic pain. On the other hand, older adults with chronic pain who had hair cortisol levels from the 30th percentile and over were less likely to recover from poor mental health. Moreover, chronic pain was not associated with hair cortisol among older adults at the 30th and 40th percentiles of the cortisol distribution in the ELSA sample, and at the 20th and 60th percentiles in the UKHLS-IP sample. This suggests that moderate levels of cortisol could be an indicator of an inadequate cortisol response to chronic pain. Whereas the associations of the highest levels of hair cortisol with both chronic pain and poor mental health suggest that the highest levels of hair cortisol produced in relation to chronic pain correspond to a prolonged cortisol response. The use of the quantile regression methods enabled us to address a key contradiction in the research on the association between chronic pain and cortisol. With many studies reporting conflicting (positive, negative, or null) associations, previous analyses may have been inadequate because they did not account for negative feedback loops in the HPA axis functioning. Standard linear regression models do not describe the heterogeneity in cortisol responses to chronic pain. Future research on the analysis of stressors on measures of HPA axis functioning needs to use statistical methods that are more sensitive to differential patterns across the distribution of HPA axis functioning, such as quantile regression models.

The other key significance of this study is evidence of a biomarker associated with resilience to chronic pain. While promising candidate biomarkers for resilience have been proposed in physiological, neurochemical, and immune systems, such as blood microRNAs, cortisol, and chemokines (Chen et al., 2015; Lau et al., 2021; Walker et al., 2017; Zhang et al., 2020), their interpretation and application remain challenging due to the multifaceted nature of biological systems and the influence of various factors on biomarker levels. There has also been a lack of evidence on the predictive validity of potential candidate biomarkers of resilience in terms of predicting future ill-health. Biomarkers of the stress response can provide information about how an individual is currently responding to stress, but they may not accurately predict how that individual will respond to future stressors. Biomarkers of resilience to stress, on the other hand, can potentially provide more accurate predictions about an individual’s future stress response and future health states (Sweeten, Sutton, Wellman, & Sanford, 2020). While cortisol is a known key measure of the stress response, the analysis also demonstrated how a low cortisol response to chronic pain protects against future mental ill-health, in contrast to a null or inadequate cortisol response to chronic pain. Low cortisol levels following chronic pain may be an indicator of resilience. The measurement of hair cortisol as a potential biomarker of resilience offers a quantifiable dimension to this complex construct and also enriches our understanding of the biological foundations of resilience.

As hair cortisol is a noninvasive biomarker and is easier to collect in surveys than blood-based biomarkers, this could be an important measure to collect for surveys interested in physiological stress responses, as well as resilient responses to chronic stress. A key strength of the study was the use of data from two population representative surveys, with similar patterns of association with measurements of hair cortisol conducted from the same lab. However, this leads us to the key limitation of the study, which is that hair cortisol could not be collected in a large proportion of the sample (between 20 and 25% of the eligible sample). In particular, older men and women in the ELSA study were more likely to be missing a hair sample compared to younger men. Existing research on missing data analyses of the ELSA hair cortisol samples suggests that IPW is the preferred missing data compensation method (Chatzi et al., 2024). In addition, the use of differing measures of mental health across surveys introduces a degree of variability that may affect the comparability of the results. However, the consistency in patterns observed across both studies mitigates this concern. The prediction analyses assumed that chronic pain predicts future mental ill-health, although depression and painful symptoms commonly occur together. Hence, it was important to restrict the prediction analyses only to those with chronic pain/pain interference at baseline and also to distinguish between the incidence and recurrence of mental ill-health (Bair, Robinson, Katon, & Kroenke, 2003).

Hair cortisol can be used not only as a measure of chronic stress response among individuals in population surveys, but also as a measure that can identify resilient individuals to chronic pain. Surveys interested in measuring physiological stress responses should consider measuring hair cortisol as a noninvasive objective marker of the stress response and a potential indicator of resiliency to chronic stress. Future research that uses biomarkers related to stress needs to use appropriate statistical models that explore the effects across the distributions of the biomarkers.

## Supporting information

Chandola et al. supplementary material Chandola et al. supplementary material

## Supplementary material

The supplementary material for this article can be found at http://doi.org/10.1017/S0033291725101049.

## Classification

Major: Social Sciences; Minor: Psychological and Cognitive Science.
