# Supplementary material for: Measuring resilience to chronic pain in population surveys using hair cortisol
Source: Psychol Med. 2025 Aug 15;55:e201. doi: 10.1017/S0033291725101049 (PMC12404322; doi:10.1017/S0033291725101049)
Supplement: Chandola et al. supplementary material [file S0033291725101049sup001.docx]

**Supplementary Appendix**

Measuring resilience to chronic pain in longitudinal population studies using hair cortisol

**This file includes:**

Figures S1 to S2

Tables S1 to S5

Description of the datasets and the hair sample collection process

Description of analytical approaches

**Steps of the analytical sample construction**

Data from ELSA w6 (n = 10,601)

Excluding:

2,547 participants who were not taking part in the nurse visit (n = 8,054)

Participants who involved in the nurse visit (n = 8,054)

Excluding:

1,989 participants who pregnancy, breastfeeding, scalp conditions, inability to maintain a steady head position, and less than 2 cm of hair length in the posterior vertex area (n = 6,061)

555 refusals (n=5,506)

55 participants unable to obtain hair (n=5,451)

123 participants failed to extract steroid hormones (n=5,328)

Participants successfully extracted steroid hormones (n=5,328)

Excluding:

417 participants without detectible cortisol values (n = 4,911)

83 participants with cortisol above 660 pg/ml (n = 4,828)

184 participants without nurse visit weight (n = 4,644)

Participants without missing values for cortisol (n = 4,644)

Excluding:

20 participants with missing education levels (n = 4,624)

Participants without missing socio-demographic characteristics (n = 4,624)

Excluding:

50 participants with missing CESD-8 depression at ELSA w6 (n = 4,575)

1,349 participants with missing CESD-8 depression at ELSA w9 (n =3,226)

Excluding:

41 participants with missing information for the survey season (n = 4,583)

23 participants without missing hair colour and chemically treated information (n = 4,560)

Analytical sample for estimating the association between chronic pain and cortisol at different quantiles (n = 4,560)

Analytical sample for predicting cases of mental ill-health among older adults (n =3,226)

Figure S1. Flowchart for the analytical sample construction: ELSA

**Steps of the analytical sample construction**

Data from UKHLS -IP w12 (n = 2,267)

Excluding:

1,353 participants who did not provide hair samples (n = 914)

Participants who provided hair samples (n = 914)

Excluding:

64 participants without detectible cortisol values (n = 850)

Participants without missing values for cortisol (n = 850)

Excluding:

62 participants with missing pain levels (n = 788)

258 participants aged 50 years and above (n = 530)

Participants without missing socio-demographic characteristics (n = 530)

Excluding:

57 participants without missing hair chemically treated information (n = 473)

Excluding:

12 participants with missing GHQ depression at UKHLS -IP w12 (n = 518)

47 participants with missing GHQ depression at UKHLS -IP w13 (n =471)

Analytical sample for estimating the association between pain and cortisol at different quantiles (n = 473)

Analytical sample for predicting cases of mental ill-health among older adults (n =471)

Figure S2. Flowchart for the analytical sample construction: UKHLS-IP

Table S1. Cross-sectional quantile regression model examining the association between chronic pain level and (log) cortisol quantile: ELSA w6.

|  | (log) cortisol | |
| --- | --- | --- |
|  | Coef. | CI |
| Quantile 10 |  |  |
| Chronic pain (Ref.= No severe) |  |  |
| Moderate/Severe pain | 0.16^***^ | [0.08,0.25] |
| Sex (Ref. = Male) |  |  |
| Female | -0.1 | [-0.20,0.00] |
| Age group (Ref. = 50-54) |  |  |
| 55-59 | 0.16^*^ | [0.03,0.30] |
| 60-64 | 0.09 | [-0.05,0.23] |
| 65-69 | 0.20^**^ | [0.05,0.34] |
| 70-74 | 0.16 | [-0.00,0.32] |
| 75-79 | 0.08 | [-0.09,0.25] |
| 80+ | 0.25^**^ | [0.09,0.41] |
| Number of medications (Ref.= None) |  |  |
| Once | 0.09 | [-0.03,0.21] |
| Twice | 0.04 | [-0.08,0.17] |
| Three times | -0.01 | [-0.15,0.13] |
| Four times and more | 0.04 | [-0.07,0.14] |
| Ethnicity (Ref. = White) |  |  |
| Non-white | -0.22^*^ | [-0.43,-0.01] |
| Educational level (Ref.= No qualification) |  |  |
| Higher education and above | 0 | [-0.12,0.12] |
| Higher education below | 0 | [-0.10,0.09] |
| Foreign or others | -0.04 | [-0.17,0.09] |
| Relationship status (Ref.= Other) |  |  |
| Married | -0.07 | [-0.15,0.02] |
| Cohabit | 0.08 | [-0.09,0.25] |
| Whether smoking (Ref. = Never smoked) |  |  |
| Ever smoked | -0.04 | [-0.12,0.04] |
| Current smoker | 0.07 | [-0.05,0.18] |
| Hair colour (Ref. = Grey) |  |  |
| Brown | 0.12^*^ | [0.02,0.23] |
| Blonde | -0.19^**^ | [-0.32,-0.06] |
| Other | 0.08 | [-0.02,0.18] |
| Whether dye or chemically treated hair (Ref. = No) |  |  |
| Yes | -0.13^**^ | [-0.23,-0.03] |
| Phase (Ref. = Phase 1) |  |  |
| Phase 2 | 0.27^***^ | [0.19,0.36] |
| Season (Ref. = Spring) |  |  |
| Winter | -0.26^**^ | [-0.42,-0.10] |
| Summer | -0.20^*^ | [-0.36,-0.04] |
| Autumn | -0.19^*^ | [-0.34,-0.03] |
| Constant | 0.68^***^ | [0.44,0.91] |
| Quantile 20 |  |  |
| Chronic pain (Ref.= No severe) |  |  |
| Moderate/Severe pain | 0.10^**^ | [0.03,0.17] |
| Sex (Ref. = Male) |  |  |
| Female | -0.12^**^ | [-0.21,-0.03] |
| Age group (Ref. = 50-54) |  |  |
| 55-59 | 0.15^*^ | [0.03,0.27] |
| 60-64 | 0.05 | [-0.08,0.17] |
| 65-69 | 0.12 | [-0.01,0.25] |
| 70-74 | 0.15^*^ | [0.01,0.30] |
| 75-79 | 0.12 | [-0.04,0.27] |
| 80+ | 0.22^**^ | [0.08,0.37] |
| Number of medications (Ref.= None) |  |  |
| Once | -0.01 | [-0.12,0.10] |
| Twice | -0.04 | [-0.15,0.07] |
| Three times | 0.01 | [-0.11,0.14] |
| Four times and more | 0.04 | [-0.05,0.13] |
| Ethnicity (Ref. = White) |  |  |
| Non-white | -0.17 | [-0.35,0.02] |
| Educational level (Ref.= No qualification) |  |  |
| Higher education and above | 0.1 | [-0.01,0.21] |
| Higher education below | 0.02 | [-0.06,0.10] |
| Foreign or others | 0.03 | [-0.09,0.14] |
| Relationship status (Ref.= Other) |  |  |
| Married | -0.04 | [-0.11,0.04] |
| Cohabit | 0.12 | [-0.03,0.27] |
| Whether smoking (Ref. = Never smoked) |  |  |
| Ever smoked | 0.01 | [-0.06,0.08] |
| Current smoker | 0.11^*^ | [0.01,0.22] |
| Hair colour (Ref. = Grey) |  |  |
| Brown | 0.18^***^ | [0.09,0.28] |
| Blonde | -0.1 | [-0.21,0.02] |
| Other | 0.12^**^ | [0.03,0.22] |
| Whether dye or chemically treated hair (Ref. = No) |  |  |
| Yes | -0.08 | [-0.17,0.01] |
| Phase (Ref. = Phase 1) |  |  |
| Phase 2 | 0.20^***^ | [0.13,0.28] |
| Season (Ref. = Spring) |  |  |
| Winter | -0.1 | [-0.25,0.04] |
| Summer | 0 | [-0.14,0.15] |
| Autumn | 0.01 | [-0.12,0.15] |
| Constant | 0.78^***^ | [0.57,0.99] |
| Quantile 30 |  |  |
| Chronic pain (Ref.= No severe) |  |  |
| Moderate/Severe pain | 0.06 | [-0.02,0.13] |
| Sex (Ref. = Male) |  |  |
| Female | -0.13^**^ | [-0.22,-0.04] |
| Age group (Ref. = 50-54) |  |  |
| 55-59 | 0.11 | [-0.01,0.23] |
| 60-64 | 0.07 | [-0.05,0.20] |
| 65-69 | 0.12 | [-0.01,0.25] |
| 70-74 | 0.19^**^ | [0.05,0.34] |
| 75-79 | 0.18^*^ | [0.03,0.34] |
| 80+ | 0.23^**^ | [0.08,0.37] |
| Number of medications (Ref.= None) |  |  |
| Once | 0.01 | [-0.10,0.12] |
| Twice | -0.03 | [-0.14,0.08] |
| Three times | 0.03 | [-0.09,0.16] |
| Four times and more | 0.12^*^ | [0.03,0.21] |
| Ethnicity (Ref. = White) |  |  |
| Non-white | -0.28^**^ | [-0.46,-0.09] |
| Educational level (Ref.= No qualification) |  |  |
| Higher education and above | 0.11^*^ | [0.00,0.22] |
| Higher education below | 0.04 | [-0.04,0.13] |
| Foreign or others | 0.09 | [-0.03,0.20] |
| Relationship status (Ref.= Other) |  |  |
| Married | 0.04 | [-0.04,0.11] |
| Cohabit | 0.17^*^ | [0.02,0.33] |
| Whether smoking (Ref. = Never smoked) |  |  |
| Ever smoked | 0 | [-0.07,0.07] |
| Current smoker | 0.09 | [-0.01,0.20] |
| Hair colour (Ref. = Grey) |  |  |
| Brown | 0.15^**^ | [0.06,0.25] |
| Blonde | -0.09 | [-0.21,0.02] |
| Other | 0.11^*^ | [0.01,0.20] |
| Whether dye or chemically treated hair (Ref. = No) |  |  |
| Yes | -0.03 | [-0.12,0.06] |
| Phase (Ref. = Phase 1) |  |  |
| Phase 2 | 0.15^***^ | [0.08,0.23] |
| Season (Ref. = Spring) |  |  |
| Winter | -0.05 | [-0.20,0.09] |
| Summer | 0.04 | [-0.11,0.18] |
| Autumn | 0.09 | [-0.05,0.22] |
| Constant | 0.91^***^ | [0.70,1.12] |
| Quantile 40 |  |  |
| Chronic pain (Ref.= No severe) |  |  |
| Moderate/Severe pain | 0.03 | [-0.05,0.11] |
| Sex (Ref. = Male) |  |  |
| Female | -0.14^**^ | [-0.23,-0.04] |
| Age group (Ref. = 50-54) |  |  |
| 55-59 | 0.07 | [-0.06,0.20] |
| 60-64 | 0.06 | [-0.07,0.20] |
| 65-69 | 0.07 | [-0.07,0.21] |
| 70-74 | 0.06 | [-0.09,0.22] |
| 75-79 | 0.06 | [-0.11,0.23] |
| 80+ | 0.09 | [-0.07,0.25] |
| Number of medications (Ref.= None) |  |  |
| Once | 0.04 | [-0.08,0.16] |
| Twice | 0.01 | [-0.11,0.13] |
| Three times | 0.08 | [-0.05,0.21] |
| Four times and more | 0.22^***^ | [0.12,0.32] |
| Ethnicity (Ref. = White) |  |  |
| Non-white | -0.09 | [-0.29,0.11] |
| Educational level (Ref.= No qualification) |  |  |
| Higher education and above | 0.03 | [-0.09,0.14] |
| Higher education below | 0 | [-0.09,0.09] |
| Foreign or others | 0.05 | [-0.07,0.18] |
| Relationship status (Ref.= Other) |  |  |
| Married | 0.02 | [-0.06,0.11] |
| Cohabit | -0.02 | [-0.18,0.15] |
| Whether smoking (Ref. = Never smoked) |  |  |
| Ever smoked | 0.04 | [-0.04,0.11] |
| Current smoker | 0.1 | [-0.01,0.22] |
| Hair colour (Ref. = Grey) |  |  |
| Brown | 0.08 | [-0.02,0.18] |
| Blonde | -0.08 | [-0.20,0.05] |
| Other | 0.09 | [-0.01,0.19] |
| Whether dye or chemically treated hair (Ref. = No) |  |  |
| Yes | -0.03 | [-0.13,0.06] |
| Phase (Ref. = Phase 1) |  |  |
| Phase 2 | 0.03 | [-0.06,0.11] |
| Season (Ref. = Spring) |  |  |
| Winter | -0.05 | [-0.21,0.10] |
| Summer | 0.14 | [-0.01,0.30] |
| Autumn | 0.12 | [-0.03,0.27] |
| Constant | 1.23^***^ | [1.00,1.46] |
| Quantile 50 |  |  |
| Chronic pain (Ref.= No severe) |  |  |
| Moderate/Severe pain | 0.11^*^ | [0.01,0.20] |
| Sex (Ref. = Male) |  |  |
| Female | -0.15^**^ | [-0.26,-0.05] |
| Age group (Ref. = 50-54) |  |  |
| 55-59 | 0.12 | [-0.03,0.26] |
| 60-64 | 0.04 | [-0.12,0.19] |
| 65-69 | 0.04 | [-0.13,0.20] |
| 70-74 | 0.01 | [-0.17,0.18] |
| 75-79 | 0.03 | [-0.16,0.22] |
| 80+ | 0 | [-0.18,0.18] |
| Number of medications (Ref.= None) |  |  |
| Once | 0.03 | [-0.11,0.16] |
| Twice | 0.06 | [-0.08,0.19] |
| Three times | 0.15 | [-0.01,0.30] |
| Four times and more | 0.32^***^ | [0.21,0.44] |
| Ethnicity (Ref. = White) |  |  |
| Non-white | -0.1 | [-0.33,0.13] |
| Educational level (Ref.= No qualification) |  |  |
| Higher education and above | -0.06 | [-0.20,0.07] |
| Higher education below | -0.05 | [-0.16,0.05] |
| Foreign or others | 0.01 | [-0.14,0.15] |
| Relationship status (Ref.= Other) |  |  |
| Married | 0.01 | [-0.08,0.11] |
| Cohabit | -0.09 | [-0.28,0.10] |
| Whether smoking (Ref. = Never smoked) |  |  |
| Ever smoked | 0.09 | [-0.00,0.17] |
| Current smoker | 0.15^*^ | [0.02,0.28] |
| Hair colour (Ref. = Grey) |  |  |
| Brown | 0.13^*^ | [0.02,0.25] |
| Blonde | -0.06 | [-0.21,0.08] |
| Other | 0.09 | [-0.02,0.21] |
| Whether dye or chemically treated hair (Ref. = No) |  |  |
| Yes | 0.01 | [-0.10,0.12] |
| Phase (Ref. = Phase 1) |  |  |
| Phase 2 | -0.09 | [-0.18,0.00] |
| Season (Ref. = Spring) |  |  |
| Winter | -0.15 | [-0.33,0.03] |
| Summer | 0.18 | [-0.00,0.36] |
| Autumn | 0.05 | [-0.12,0.22] |
| Constant | 1.55^***^ | [1.29,1.81] |
| Quantile 60 |  |  |
| Chronic pain (Ref.= No severe) |  |  |
| Moderate/Severe pain | 0.19^***^ | [0.08,0.30] |
| Sex (Ref. = Male) |  |  |
| Female | -0.19^**^ | [-0.32,-0.06] |
| Age group (Ref. = 50-54) |  |  |
| 55-59 | 0.06 | [-0.12,0.23] |
| 60-64 | 0.01 | [-0.17,0.20] |
| 65-69 | -0.03 | [-0.22,0.16] |
| 70-74 | -0.09 | [-0.30,0.13] |
| 75-79 | 0.02 | [-0.21,0.25] |
| 80+ | -0.06 | [-0.28,0.16] |
| Number of medications (Ref.= None) |  |  |
| Once | 0.06 | [-0.10,0.22] |
| Twice | 0.15 | [-0.02,0.31] |
| Three times | 0.27^**^ | [0.09,0.46] |
| Four times and more | 0.40^***^ | [0.26,0.53] |
| Ethnicity (Ref. = White) |  |  |
| Non-white | -0.05 | [-0.33,0.22] |
| Educational level (Ref.= No qualification) |  |  |
| Higher education and above | -0.09 | [-0.26,0.07] |
| Higher education below | 0 | [-0.12,0.12] |
| Foreign or others | 0.01 | [-0.17,0.18] |
| Relationship status (Ref.= Other) |  |  |
| Married | 0.06 | [-0.06,0.17] |
| Cohabit | -0.29^*^ | [-0.52,-0.07] |
| Whether smoking (Ref. = Never smoked) |  |  |
| Ever smoked | 0.11^*^ | [0.00,0.22] |
| Current smoker | 0.22^**^ | [0.06,0.38] |
| Hair colour (Ref. = Grey) |  |  |
| Brown | 0.22^**^ | [0.09,0.36] |
| Blonde | 0.01 | [-0.16,0.18] |
| Other | 0.09 | [-0.05,0.23] |
| Whether dye or chemically treated hair (Ref. = No) |  |  |
| Yes | 0.01 | [-0.12,0.14] |
| Phase (Ref. = Phase 1) |  |  |
| Phase 2 | -0.24^***^ | [-0.36,-0.13] |
| Season (Ref. = Spring) |  |  |
| Winter | -0.04 | [-0.26,0.17] |
| Summer | 0.23^*^ | [0.02,0.45] |
| Autumn | 0.13 | [-0.07,0.34] |
| Constant | 1.75^***^ | [1.44,2.07] |
| Quantile 70 |  |  |
| Chronic pain (Ref.= No severe) |  |  |
| Moderate/Severe pain | 0.22^**^ | [0.08,0.35] |
| Sex (Ref. = Male) |  |  |
| Female | -0.1 | [-0.27,0.06] |
| Age group (Ref. = 50-54) |  |  |
| 55-59 | -0.06 | [-0.27,0.16] |
| 60-64 | -0.12 | [-0.35,0.11] |
| 65-69 | -0.13 | [-0.37,0.11] |
| 70-74 | -0.23 | [-0.49,0.04] |
| 75-79 | -0.13 | [-0.41,0.15] |
| 80+ | -0.15 | [-0.42,0.12] |
| Number of medications (Ref.= None) |  |  |
| Once | 0.14 | [-0.06,0.34] |
| Twice | 0.34^**^ | [0.13,0.54] |
| Three times | 0.37^**^ | [0.15,0.60] |
| Four times and more | 0.50^***^ | [0.33,0.67] |
| Ethnicity (Ref. = White) |  |  |
| Non-white | -0.35^*^ | [-0.69,-0.00] |
| Educational level (Ref.= No qualification) |  |  |
| Higher education and above | -0.12 | [-0.32,0.07] |
| Higher education below | 0 | [-0.15,0.15] |
| Foreign or others | 0.04 | [-0.17,0.25] |
| Relationship status (Ref.= Other) |  |  |
| Married | -0.01 | [-0.15,0.13] |
| Cohabit | -0.32^*^ | [-0.60,-0.04] |
| Whether smoking (Ref. = Never smoked) |  |  |
| Ever smoked | 0.03 | [-0.10,0.16] |
| Current smoker | 0.13 | [-0.06,0.32] |
| Hair colour (Ref. = Grey) |  |  |
| Brown | 0.16 | [-0.01,0.33] |
| Blonde | 0.01 | [-0.20,0.23] |
| Other | 0.03 | [-0.14,0.20] |
| Whether dye or chemically treated hair (Ref. = No) |  |  |
| Yes | -0.07 | [-0.23,0.09] |
| Phase (Ref. = Phase 1) |  |  |
| Phase 2 | -0.46^***^ | [-0.59,-0.32] |
| Season (Ref. = Spring) |  |  |
| Winter | -0.12 | [-0.38,0.14] |
| Summer | 0.14 | [-0.13,0.40] |
| Autumn | 0.01 | [-0.24,0.26] |
| Constant | 2.47^***^ | [2.08,2.86] |
| Quantile 80 |  |  |
| Chronic pain (Ref.= No severe) |  |  |
| Moderate/Severe pain | 0.22^*^ | [0.05,0.39] |
| Sex (Ref. = Male) |  |  |
| Female | -0.02 | [-0.22,0.18] |
| Age group (Ref. = 50-54) |  |  |
| 55-59 | 0 | [-0.27,0.27] |
| 60-64 | -0.14 | [-0.42,0.15] |
| 65-69 | 0.05 | [-0.25,0.35] |
| 70-74 | -0.08 | [-0.41,0.24] |
| 75-79 | 0.01 | [-0.34,0.36] |
| 80+ | 0.08 | [-0.25,0.42] |
| Number of medications (Ref.= None) |  |  |
| Once | 0.06 | [-0.19,0.30] |
| Twice | 0.24 | [-0.01,0.50] |
| Three times | 0.29^*^ | [0.01,0.57] |
| Four times and more | 0.42^***^ | [0.21,0.63] |
| Ethnicity (Ref. = White) |  |  |
| Non-white | -0.38 | [-0.80,0.05] |
| Educational level (Ref.= No qualification) |  |  |
| Higher education and above | -0.17 | [-0.42,0.08] |
| Higher education below | -0.06 | [-0.25,0.12] |
| Foreign or others | -0.01 | [-0.27,0.25] |
| Relationship status (Ref.= Other) |  |  |
| Married | 0.08 | [-0.08,0.25] |
| Cohabit | -0.27 | [-0.62,0.08] |
| Whether smoking (Ref. = Never smoked) |  |  |
| Ever smoked | 0.05 | [-0.12,0.21] |
| Current smoker | 0.11 | [-0.13,0.35] |
| Hair colour (Ref. = Grey) |  |  |
| Brown | -0.06 | [-0.27,0.15] |
| Blonde | -0.15 | [-0.41,0.11] |
| Other | -0.03 | [-0.24,0.18] |
| Whether dye or chemically treated hair (Ref. = No) |  |  |
| Yes | 0.05 | [-0.15,0.25] |
| Phase (Ref. = Phase 1) |  |  |
| Phase 2 | -0.58^***^ | [-0.75,-0.41] |
| Season (Ref. = Spring) |  |  |
| Winter | -0.18 | [-0.51,0.14] |
| Summer | 0.06 | [-0.27,0.38] |
| Autumn | -0.21 | [-0.52,0.10] |
| Constant | 3.13^***^ | [2.65,3.61] |
| Quantile 90 |  |  |
| Chronic pain (Ref.= No severe) |  |  |
| Moderate/Severe pain | 0.21 | [-0.09,0.50] |
| Sex (Ref. = Male) |  |  |
| Female | 0.1 | [-0.25,0.45] |
| Age group (Ref. = 50-54) |  |  |
| 55-59 | -0.48^*^ | [-0.95,-0.00] |
| 60-64 | -0.66^*^ | [-1.16,-0.16] |
| 65-69 | -0.56^*^ | [-1.08,-0.04] |
| 70-74 | -0.68^*^ | [-1.25,-0.11] |
| 75-79 | -0.55 | [-1.16,0.07] |
| 80+ | -0.15 | [-0.74,0.43] |
| Number of medications (Ref.= None) |  |  |
| Once | 0.24 | [-0.20,0.67] |
| Twice | 0.11 | [-0.33,0.56] |
| Three times | 0.25 | [-0.24,0.75] |
| Four times and more | 0.43^*^ | [0.06,0.80] |
| Ethnicity (Ref. = White) |  |  |
| Non-white | -0.52 | [-1.27,0.22] |
| Educational level (Ref.= No qualification) |  |  |
| Higher education and above | -0.27 | [-0.71,0.16] |
| Higher education below | -0.22 | [-0.55,0.11] |
| Foreign or others | -0.09 | [-0.56,0.37] |
| Relationship status (Ref.= Other) |  |  |
| Married | -0.14 | [-0.44,0.16] |
| Cohabit | -0.33 | [-0.94,0.28] |
| Whether smoking (Ref. = Never smoked) |  |  |
| Ever smoked | -0.06 | [-0.35,0.22] |
| Current smoker | -0.17 | [-0.58,0.25] |
| Hair colour (Ref. = Grey) |  |  |
| Brown | -0.21 | [-0.58,0.16] |
| Blonde | -0.46^*^ | [-0.92,-0.00] |
| Other | -0.13 | [-0.50,0.24] |
| Whether dye or chemically treated hair (Ref. = No) |  |  |
| Yes | 0.1 | [-0.25,0.45] |
| Phase (Ref. = Phase 1) |  |  |
| Phase 2 | -1.03^***^ | [-1.33,-0.73] |
| Season (Ref. = Spring) |  |  |
| Winter | -0.42 | [-0.99,0.16] |
| Summer | -0.48 | [-1.06,0.10] |
| Autumn | -0.59^*^ | [-1.14,-0.04] |
| Constant | 5.44^***^ | [4.60,6.28] |
| N(observations) | 4560 |  |
| *Note*. 95% confidence intervals in brackets. * p < 0.05, ** p < 0.01, *** p < 0.001 | | |

Table S2. Cross-sectional quantile regression model examining the association between pain level and (log) cortisol quantile: UKHLS-IP w12.

|  | (log) cortisol | |
| --- | --- | --- |
|  | Coef. | CI |
| Quantile 10 |  |  |
| Pain (Ref. = No severe) |  |  |
| Quite a bit/Extreme interference | 0.38^*^ | [0.04,0.73] |
| Age group (Ref. = 50-54) |  |  |
| 55-59 | -0.17 | [-0.54,0.20] |
| 60-64 | -0.05 | [-0.42,0.32] |
| 65-69 | 0.05 | [-0.32,0.43] |
| 70-74 | -0.08 | [-0.46,0.30] |
| 75-78 | 0.08 | [-0.38,0.54] |
| 80+ | -0.18 | [-0.65,0.30] |
| Sex (Ref. = Male) |  |  |
| Female | -0.28^*^ | [-0.55,-0.00] |
| Hair treated (Ref.= No) |  |  |
| Yes | -0.34^*^ | [-0.62,-0.06] |
| Constant | 1.15^***^ | [0.86,1.45] |
| Quantile 20 |  |  |
| Pain (Ref. = No severe) |  |  |
| Quite a bit/Extreme interference | 0.26 | [-0.09,0.61] |
| Age group (Ref. = 50-54) |  |  |
| 55-59 | -0.15 | [-0.52,0.22] |
| 60-64 | -0.23 | [-0.60,0.15] |
| 65-69 | 0.03 | [-0.35,0.41] |
| 70-74 | -0.19 | [-0.57,0.19] |
| 75-78 | 0.06 | [-0.41,0.52] |
| 80+ | -0.21 | [-0.69,0.27] |
| Sex (Ref. = Male) |  |  |
| Female | -0.29^*^ | [-0.57,-0.02] |
| Hair treated (Ref.= No) |  |  |
| Yes | -0.19 | [-0.47,0.10] |
| Constant | 1.50^***^ | [1.20,1.80] |
| Quantile 30 |  |  |
| Pain (Ref. = No severe) |  |  |
| Quite a bit/Extreme interference | 0.17 | [-0.18,0.53] |
| Age group (Ref. = 50-54) |  |  |
| 55-59 | -0.27 | [-0.64,0.11] |
| 60-64 | -0.52^**^ | [-0.89,-0.14] |
| 65-69 | -0.11 | [-0.49,0.28] |
| 70-74 | -0.25 | [-0.63,0.14] |
| 75-78 | -0.18 | [-0.65,0.30] |
| 80+ | -0.04 | [-0.53,0.45] |
| Sex (Ref. = Male) |  |  |
| Female | -0.19 | [-0.47,0.09] |
| Hair treated (Ref.= No) |  |  |
| Yes | -0.25 | [-0.54,0.04] |
| Constant | 1.85^***^ | [1.54,2.15] |
| Quantile 40 |  |  |
| Pain (Ref. = No severe) |  |  |
| Quite a bit/Extreme interference | 0.29 | [-0.07,0.64] |
| Age group (Ref. = 50-54) |  |  |
| 55-59 | -0.24 | [-0.62,0.14] |
| 60-64 | -0.33 | [-0.71,0.04] |
| 65-69 | -0.02 | [-0.40,0.37] |
| 70-74 | -0.09 | [-0.48,0.29] |
| 75-78 | -0.49^*^ | [-0.96,-0.01] |
| 80+ | -0.23 | [-0.72,0.26] |
| Sex (Ref. = Male) |  |  |
| Female | -0.35^*^ | [-0.63,-0.07] |
| Hair treated (Ref.= No) |  |  |
| Yes | -0.12 | [-0.41,0.17] |
| Constant | 2.11^***^ | [1.81,2.42] |
| Quantile 50 |  |  |
| Pain (Ref. = No severe) |  |  |
| Quite a bit/Extreme interference | 0.33 | [-0.04,0.70] |
| Age group (Ref. = 50-54) |  |  |
| 55-59 | 0.03 | [-0.36,0.42] |
| 60-64 | -0.31 | [-0.70,0.08] |
| 65-69 | -0.06 | [-0.46,0.34] |
| 70-74 | 0.1 | [-0.29,0.50] |
| 75-78 | -0.4 | [-0.89,0.09] |
| 80+ | -0.04 | [-0.54,0.47] |
| Sex (Ref. = Male) |  |  |
| Female | -0.43^**^ | [-0.72,-0.14] |
| Hair treated (Ref.= No) |  |  |
| Yes | 0 | [-0.30,0.30] |
| Constant | 2.22^***^ | [1.90,2.53] |
| Quantile 60 |  |  |
| Pain (Ref. = No severe) |  |  |
| Quite a bit/Extreme interference | 0.29 | [-0.11,0.70] |
| Age group (Ref. = 50-54) |  |  |
| 55-59 | 0.12 | [-0.31,0.55] |
| 60-64 | -0.39 | [-0.82,0.04] |
| 65-69 | -0.1 | [-0.54,0.34] |
| 70-74 | -0.1 | [-0.53,0.34] |
| 75-78 | -0.62^*^ | [-1.16,-0.08] |
| 80+ | -0.07 | [-0.63,0.49] |
| Sex (Ref. = Male) |  |  |
| Female | -0.39^*^ | [-0.71,-0.07] |
| Hair treated (Ref.= No) |  |  |
| Yes | -0.16 | [-0.49,0.17] |
| Constant | 2.58^***^ | [2.23,2.93] |
| Quantile 70 |  |  |
| Pain (Ref. = No severe) |  |  |
| Quite a bit/Extreme interference | 0.51^*^ | [0.02,1.00] |
| Age group (Ref. = 50-54) |  |  |
| 55-59 | 0.07 | [-0.45,0.59] |
| 60-64 | -0.49 | [-1.01,0.03] |
| 65-69 | -0.18 | [-0.72,0.35] |
| 70-74 | -0.08 | [-0.61,0.45] |
| 75-78 | -0.48 | [-1.13,0.17] |
| 80+ | -0.05 | [-0.72,0.62] |
| Sex (Ref. = Male) |  |  |
| Female | -0.64^**^ | [-1.03,-0.25] |
| Hair treated (Ref.= No) |  |  |
| Yes | -0.08 | [-0.48,0.31] |
| Constant | 3.01^***^ | [2.59,3.43] |
| Quantile 80 |  |  |
| Pain (Ref. = No severe) |  |  |
| Quite a bit/Extreme interference | 0.65 | [-0.00,1.30] |
| Age group (Ref. = 50-54) |  |  |
| 55-59 | -0.15 | [-0.84,0.54] |
| 60-64 | -0.78^*^ | [-1.46,-0.09] |
| 65-69 | -0.52 | [-1.23,0.18] |
| 70-74 | -0.23 | [-0.93,0.48] |
| 75-78 | -0.6 | [-1.47,0.26] |
| 80+ | -0.63 | [-1.53,0.26] |
| Sex (Ref. = Male) |  |  |
| Female | -0.75^**^ | [-1.26,-0.23] |
| Hair treated (Ref.= No) |  |  |
| Yes | -0.06 | [-0.59,0.47] |
| Constant | 3.80^***^ | [3.24,4.35] |
| Quantile 90 |  |  |
| Pain (Ref. = No severe) |  |  |
| Quite a bit/Extreme interference | 1.30^**^ | [0.33,2.27] |
| Age group (Ref. = 50-54) |  |  |
| 55-59 | -0.71 | [-1.73,0.32] |
| 60-64 | -0.59 | [-1.62,0.44] |
| 65-69 | -0.03 | [-1.09,1.03] |
| 70-74 | -0.63 | [-1.68,0.42] |
| 75-78 | -0.16 | [-1.45,1.13] |
| 80+ | -0.42 | [-1.75,0.92] |
| Sex (Ref. = Male) |  |  |
| Female | 0.05 | [-0.72,0.81] |
| Hair treated (Ref.= No) |  |  |
| Yes | -0.57 | [-1.35,0.22] |
| Constant | 4.26^***^ | [3.42,5.09] |
| N(observations) | 473 |  |
| *Note.* 95% confidence intervals in brackets. * p < 0.05, ** p < 0.01, *** p < 0.001 | | |

Table S3. Logistic regression model examining the effect of interactions between chronic pain, CESD-8 depression at ELSA w6 and (log) cortisol level in predicting CESD-8 depression at w9.

|  | CESD-8 at ELSA w9 | |
| --- | --- | --- |
|  | Coef. | CI |
| Chronic pain (Ref.= No severe) |  |  |
| Moderate/Severe pain | 1.05^***^ | [0.71,1.39] |
| CESD-8 at w6 (Ref. = CESD-8 score < 4) |  |  |
| CESD-8 score >= 4 | 2.45^***^ | [2.02,2.89] |
| (log) cortisol | -0.16 | [-0.36,0.05] |
| Chronic pain * CESD-8 at w6 |  |  |
| Moderate/Severe pain * CESD-8 score >= 4 | -0.56 | [-1.20,0.07] |
| Chronic pain * (log) cortisol |  |  |
| Moderate/Severe pain * (log) cortisol | -0.06 | [-0.36,0.25] |
| CESD-8 at w6 * (log) cortisol |  |  |
| CESD-8 score >= 4 * (log) cortisol | -0.06 | [-0.47,0.34] |
| Chronic pain * CESD-8 at w6 * (log) cortisol |  |  |
| Moderate/Severe pain * CESD-8 score >= 4 * (log) cortisol | 0.83^**^ | [0.22,1.45] |
| Sex (Ref. = Female) |  |  |
| Female | 0.46^**^ | [0.13,0.79] |
| Age group (Ref. = 50-54) |  |  |
| 55-59 | -0.32 | [-0.85,0.21] |
| 60-64 | -0.23 | [-0.74,0.29] |
| 65-69 | -0.46 | [-0.99,0.07] |
| 70-74 | 0.25 | [-0.32,0.81] |
| 75-79 | 0.25 | [-0.33,0.84] |
| 80+ | 0.22 | [-0.44,0.88] |
| Educational level (Ref.= No qualification) |  |  |
| Higher education and above | -0.32 | [-0.79,0.14] |
| Higher education below | 0.1 | [-0.23,0.43] |
| Foreign or others | -0.07 | [-0.58,0.43] |
| Whether smoking (Ref. = Never smoked) |  |  |
| Ever smoked | -0.09 | [-0.40,0.23] |
| Current smoker | 0.56^*^ | [0.12,1.00] |
| Relationship status (Ref. = Neither) |  |  |
| Married | -0.38^*^ | [-0.68,-0.07] |
| Cohabit | -0.06 | [-0.69,0.57] |
| Ethnicity (Ref. = White) |  |  |
| Non-white | 1.19^***^ | [0.50,1.88] |
| Constant | -2.83^***^ | [-3.51,-2.15] |
| N(observations) | 3226 |  |
| *Note.* The (log) cortisol was standardized as z-scores. 95% confidence intervals in brackets. * p < 0.05, ** p < 0.01, *** p < 0.001 | | |

Table S4. Logistic regression model examining the effect of interactions between pain, GHQ depression at UKHLS-IP w12, and (log) cortisol level in predicting GHQ depression at w13.

|  | GHQ-caseness scores at w13 | |
| --- | --- | --- |
|  | Coef. | CI |
| Pain (Ref. = No severe) |  |  |
| Quite a bit/Extreme interference | 0.86^*^ | [0.02,1.70] |
| GHQ-caseness scores at w12 (Ref. = GHQ-12 caseness < 3) |  |  |
| GHQ caseness >= 3 | 1.00^**^ | [0.32,1.69] |
| (log) cortisol | -0.03 | [-0.31,0.25] |
| Pain * GHQ-caseness scores at w12 |  |  |
| Quite a bit/Extreme interference * GHQ caseness >= 3 | 1.01 | [-0.82,2.83] |
| Pain * (log) cortisol |  |  |
| Quite a bit/Extreme interference * (log) cortisol | -0.27 | [-1.12,0.57] |
| GHQ-caseness scores at w12 * (log) cortisol |  |  |
| GHQ caseness >= 3 * (log) cortisol | 0.45 | [-0.33,1.22] |
| Pain * GHQ-caseness scores at w12 * (log) cortisol |  |  |
| Quite a bit/Extreme interference * GHQ caseness >= 3 * (log) cortisol | 2.16^*^ | [0.10,4.23] |
| Age | -0.03^*^ | [-0.06,-0.00] |
| Sex (Ref. = Male) |  |  |
| Female | 0.34 | [-0.24,0.92] |
| Constant | 0.88 | [-1.09,2.86] |
| N(observations) | 471 |  |
| *Note.* The (log) cortisol was standardized as z-scores. 95% confidence intervals in brackets. * p < 0.05, ** p < 0.01, *** p < 0.001 | | |

# Table S5. Relevant survey questions and their order from the ELSA and UKHLS-IP questionnaires.

| **Datasets** | **Variables** | **Questions** |  |
| --- | --- | --- | --- |
| ELSA | Chronic pain | [HEPAIN]. Are you often troubled with pain? 1 - Yes 2 - No |  |
|  |  | [HEPAA]. How bad is the pain most of the time? 1 - mild, 2 - moderate 3 - or, severe |  |
|  |  | [HEPAG]. How long has the pain been bothering you?  1 - less than 3 months 2 - more than 3 months, but less than 6 months 3 - more than 6 months, but less than 12 months 4 - or more than 12 months |  |
|  | Mental ill-health | [PSCEDA]. (Much of the time during the past week), you felt depressed? 1 - Yes 2 - No |  |
|  |  | [PSCEDB]. (Much of the time during the past week), you felt that everything you did was an effort? 1 - Yes 2 - No |  |
|  |  | [PSCEDC]. (Much of the time during the past week), your sleep was restless? 1 - Yes 2 - No |  |
|  |  | [PSCEDD]. (Much of the time during the past week), you were happy? 1 - Yes 2 - No |  |
|  |  | [PSCEDE]. (Much of the time during the past week), you felt lonely? 1 - Yes 2 - No |  |
|  |  | [PSCEDF]. (Much of the time during the past week), you enjoyed life? 1 - Yes 2 - No |  |
|  |  | [PSCEDG]. (Much of the time during the past week), you felt sad? 1 - Yes 2 - No |  |
|  |  | [PSCEDH]. (Much of the time during the past week), you could not get going? 1 - Yes 2 - No |  |
|  | Sex | [DHSEXC]. Can I just check, [^is/was] [^<correct first name>] [^sex from last interview]? 1 - Yes 2 - No |  |
|  |  | [DHSEX]. Ask or code [^<correct first name>]'s sex. 1 - Male 2 - Female |  |
|  | Age | [DHDOBC]. Can I just check, [^is/was] [^<correct first name>]'s date of birth [^<date of birth from last interview>]? 1 - Yes 2 - No |  |
|  |  | [DHDOB]. What [^is/was] [^<correct first name>]'s date of birth? |  |
|  | Education level | [FQQUAL]. Which of the qualifications on this card [^do you have / have you obtained since then]? Just tell me the number written beside each one. |  |
|  |  | 1 - Degree/degree level qualification (including higher degree) 2 - Teaching qualification 3 - Nursing qualifications SRN, SCM, SEN, RGN, RM, RHV, Midwife 4 - HNC/HND, BEC/TEC Higher, BTEC Higher/SCOTECH Higher 5 - ONC/OND/BEC/TEC/BTEC not higher 6 - City and Guilds Full Technological Certificate 7 - City and Guilds Advanced/Final Level 8 - City and Guilds Craft/Ordinary Level 9 - A-levels/Higher School Certificate 10 - AS level 11 - SLC/SCE/SUPE at Higher Grade or Certificate of Sixth Year Studies 12 - O-level passes taken in 1975 or earlier 13 - O-level passes taken after 1975 GRADES A-C 14 - O-level passes taken after 1975 GRADES D-E 15 - GCSE GRADES A-C 16 - GCSE GRADES D-G 17 - CSE GRADE 1/SCE BANDS A-C/Standard Grade LEVEL 1-3 18 - CSE GRADES 2-5/SCE Ordinary BANDS D-E 19 - CSE Ungraded 20 - SLC Lower 21 - SUPE Lower or Ordinary 22 - School Certificate or Matric 23 - NVQ Level 5 24 - NVQ Level 4 25 - NVQ Level 3/Advanced level GNVQ 26 - NVQ Level 2/Intermediate level GNVQ 27 - NVQ Level 1/Foundation level GNVQ 28 - Recognised Trade Apprenticeship completed 29 - Clerical or Commercial Qualification (eg typing/book-keeping/commerce) 95 - Other qualifications |  |
|  | Relationship status | [DIMAR]. What is [^your / Name’s] current legal marital status? 1 - Single, that is never married and never registered in a same-sex Civil Partnership 2 - Married, first and only marriage 3 - A civil partner in a registered same-sexCivil Partnership 4 - Remarried, second or later marriage 5 - Separated, but still legally married 6 - Divorced 7 - Widowed 8 - Spontaneous only – Separated but still legally in a same-sex- Civil Partnership his/her civil partner 9 - Spontaneous only - Formerly in a same-sex Civil Partnership which is now legally dissolved 10 - Spontaneous only - A surviving civil partner from a same-sex Civil Partnership 11 - Spontaneous only - A civil partner in a registered same-sex Civil Partnership and has been married or in another same-sex Civil Partnership before. |  |
|  | Ethnicity | [FQETHN]. Can I check, to which of the groups on this card do you consider that [^you / [^name]] belong? 1 - White 2 - Mixed ethnic group 3 - Black 4 - Black British 5 - Asian 6 - Asian British 95 - Any other group |  |
|  | Number of medications | [DrCod1] - [DrCod27 ]. To do the drug coding now, press <Ctrl Enter>, select Drug Code with the highlight bar and press <Enter>. |  |
|  | Whether smoking | [HESMK]. [Have you/ Has [^name]] ever smoked cigarettes? 1 - Yes 2 - No |  |
|  |  | [HESKA]. [^Do you / Does [^name]] smoke cigarettes at all nowadays? 1 - Yes 2 - No |  |
|  | Hair colour | [HAIRCOL]. What colour is the hair sample? 1 - Brown 2 - Blonde 3 - Red/auburn/ginger 4 - White 5 - Grey 6 - Black 7 - Other colour (please specify) |  |
|  | Whether dye or chemically treated hair | [HAIRDYE]. Is your hair dyed? 1 - Yes 2 - No |  |
|  |  | [HAIRTREA]. Has your hair been chemically treated (eg. Perm, chemical straightening)? 1 - Yes 2 - No |  |
|  | Phase and Season | [DATEOK]. Today's date according to the laptop is [^automatically recorded date of interview]. Is this the correct date? 1 - Yes 2 - No |  |
|  |  | [NURDATE]. Enter the date of this interview. |  |
| UKHLS-IP | Chronic pain | [Scsf5]. During the past 4 weeks, how much did pain interfere with your normal work (including both work outside the home and housework)? 1 - Not at all Not at all 2 - A little bit A little bit 3 - Moderately Moderately 4 - Quite a bit Quite a bit 5 - Extremely Extremely |  |
|  |  |  |  |
|  |  |  |  |
|  | Mental ill-health | The next questions are about how you have been feeling over the last few weeks. [Scghqa]. Have you recently been able to concentrate on whatever you're doing? 1 - Better than usual Better than usual 2 - Same as usual Same as usual 3 - Less than usual Less than usual 4 - Much less than usual Much less than usual |  |
|  |  | [Scghqb]. Have you recently lost much sleep over worry? 1 - Not at all 2 - No more than usual 3 - Rather more than usual 4 - Much more than usual |  |
|  |  | [Scghqc]. Have you recently felt that you were playing a useful part in things? 1 - Not at all 2 - No more than usual 3 - Rather more than usual 4 - Much more than usual |  |
|  |  | [Scghqd]. Have you recently felt capable of making decisions about things? 1 - Not at all 2 - No more than usual 3 - Rather more than usual 4 - Much more than usual |  |
|  |  | [Scghqe]. Have you recently felt constantly under strain? 1 - Not at all 2 - No more than usual 3 - Rather more than usual 4 - Much more than usual |  |
|  |  | [Scghqf]. Have you recently felt you couldn't overcome your difficulties? 1 - Not at all 2 - No more than usual 3 - Rather more than usual 4 - Much more than usual |  |
|  |  | [Scghqg]. Have you recently been able to enjoy your normal day-to-day activities? 1 - More so than usual  2 - About the same as usual 3 - Less so than usual 4 - Much less than usual |  |
|  |  | [Scghqh]. Have you recently been able to face up to problems? 1 - More so than usual  2 - About the same as usual 3 - Less so than usual 4 - Much less than usual |  |
|  |  | [Scghqi]. Have you recently been feeling unhappy or depressed? 1 - Not at all 2 - No more than usual 3 - Rather more than usual 4 - Much more than usual |  |
|  |  | [Scghqj]. Have you recently been losing confidence in yourself? 1 - Not at all 2 - No more than usual 3 - Rather more than usual 4 - Much more than usual |  |
|  |  | [Scghqk]. Have you recently been thinking of yourself as a worthless person? 1 - Not at all 2 - No more than usual 3 - Rather more than usual 4 - Much more than usual |  |
|  |  | [Scghql]. Have you recently been feeling reasonably happy, all things considered? 1 - More so than usual  2 - About the same as usual 3 - Less so than usual 4 - Much less than usual |  |
|  | Sex | [Chkrespweb]. Can we just check, you are [NAME SURNAME] and you are [SEX] ? 1 - Yes, both correct Yes, both correct 2 - No, name is wrong No, name is wrong 3 - No, date of birth is wrong No, date of birth is wrong 4 - No, sex is wrong No, sex is wrong |  |
|  |  | [Chkwebsex]. And are you male or female? 1 - Male  2 - Female |  |
|  | Age | [Chkwebdobd]. We need to make sure we are surveying the correct person. What is your date of birth? |  |
|  |  | [Nebirthy]. What is [[your]/[NAME]'s/[ff_forname]'s] date of birth? |  |
|  | Hair treated | [hairtrea]. Had chemical hair treatment? 1 - Yes 2 - No |  |
|  |  | [hairdye]. Hair dyed? 1 - Yes 2 - No |  |
| *Note.* The order of questions (question name in the original questionnaire) is in brackets | | |  |

Description of the datasets and the hair sample collection process

***Datasets***

The English Longitudinal Study of Ageing is a longitudinal panel study of men and women aged 50 years and older living in England. The study started in 2002 and people were followed up every 2 years. The sample is periodically refreshed and comparisons of sociodemographic characteristics with the national census indicate that the sample is broadly representative of the English population. The wave 6 nurse (2012-2013) visit included a hair sample to measure cortisol, collected across two phases. Hair analysis was carried out on a subset of the 8,054 core participants who took part in wave 6 because of financial constraints. Data on hormone levels in hair were available for 5,328 individuals, of whom 4,911 had detectible cortisol values. Participants gave full informed consent and ethical approval was obtained from the National Research Ethics Service. All the analyses were restricted to ELSA participants aged 50 years and over at wave 6.

The UKHLS-Innovation Panel is a sample of 1,500 households, used as a test-bed for innovative ways of collecting data and for developing new areas of research (University Of Essex, 2023). Participants are, on the whole, asked the same questions using the same procedures as the main Understanding Society survey (University of Essex, Institute for Social and Economic Research, 2023). The UKHLS-IP is designed similar to the main survey and is a representative sample of adults living in private households in England, Scotland and Wales. There were 1,408 interviewed households at wave 12 (2019), within which 2,162 people were interviewed. Interviewees were asked to provide a sample of their hair. Participants gave full informed consent and ethical approval was obtained from the National Research Ethics Service. All the analyses were restricted to UKHLS-IP participants aged 50 years and over at wave 12, to enable comparability with the ELSA participants.

***Hair sample collection and analysis***

In ELSA, hair samples measuring a minimum of 2 cm in length and weighing at least 10 mg were gathered from the posterior vertex of consenting participants, cut as proximate to the scalp as feasible. The exclusion criteria for sample collection included pregnancy, breastfeeding, certain scalp conditions, inability to maintain a steady head position, and having less than 2 cm of hair length in the posterior vertex scalp area. Comprehensive details of the sampling procedure can be found at [ELSA project website](https://www.elsa-project.ac.uk/user-guides). Following the collection, the samples underwent a wash procedure and steroid extraction utilizing high performance liquid chromatography–mass spectrometry. Considering an average monthly hair growth of approximately 1 cm, the 2 cm scalp-adjacent hair segment represents the average cortisol accumulation over the preceding two months. Potential hair-specific factors that could influence the hair cortisol concentration—such as dyeing or chemical treatments—were evaluated via self-reporting.

In UKHLS-IP, the wave 12 included three types of interviews: nurse-led, interviewer-led, and a web-based survey. For the nurse-led interviews, participants were required to complete a consent form prior to initiating the hair sampling procedures. The collected hair samples were then dispatched to the Institute for Social and Economic Research at the University of Essex. Samples from UKHLS-IP and ELSA were sent to the [Dresden LabService GmbH](http://www.dresden-labservice.de/) for hormonal analysis. For the interviewer-led and web-based surveys, participants were presented with the consent form and a sample collection kit. The collection instructions directed participants to secure a hair sample from the posterior region close to the scalp of at least 3 cm in length and approximately 2 mm in width.

We conducted separate analyses in each dataset, to see if we obtained similar results across the datasets. In ELSA, we used data from wave 6 for a cross-sectional analysis of cortisol levels and data from wave 9 (the latest wave of data that are currently available) for a follow-up analysis of changes in poor mental health (after approximately 6 years). In UKHLS-IP, wave 12 data were utilised for a cross-sectional analysis of cortisol, while wave 13 data (the latest wave of data that are currently available) were used for the follow-up analysis of changes in poor mental health. This approach provided us with both a snapshot and a longitudinal perspective on the relationship between cortisol levels and mental ill health outcomes.

Description of analytical approaches

***Quantile regression models*** We used the Recentred Influence Function method (Firpo, Fortin, & Lemieux, 2009) to estimate the association between chronic pain and cortisol at different quantiles of the unconditional cortisol distribution ( at the 10th, 20th, 30th, 40th, 50th, 60th, 70th, 80th, 90th quantiles). The method works by applying the Recentred Influence Function (RIF) transformation at each quantile of the cortisol distribution, creating a series of binary variables for whether an observation’s outcome is above or below each quantile. Linear probability regression is then used to estimate marginal effects of covariates at each point of the distribution. Chronic pain was converted to z-statistics prior to analysis, such that coefficient represents associations between cortisol and one standard deviation increase in chronic pain. The hair cortisol values were log transformed in order to achieve a more normal distribution. As small changes in the natural log of a variable are directly interpretable as percentage changes, to a very close approximation, using the log transformed cortisol as a dependent variable in a regression model means that the estimated coefficients can be interpreted as a percentage change in cortisol.

***Non-random attrition bias*** We used inverse probability weighting (IPW) to correct the RIF and logistic regression models for potential non-random attrition bias, under the missing at random (MAR) assumption i.e. there are no unobserved predictors of missingness other than the covariates included in our model (Bhaskaran & Smeeth, 2014; Seaman & White, 2013). IPW re-weights the sample based on the probability of attrition such that characteristics of individuals who remain in the sample are identical, on average, to the full sample. Higher weights are placed on individuals who remain in the estimation samples who are more similar in characteristics to those who are lost. This results in a sample that is representative of both those lost observations and those individuals in our original sample (Seaman & White, 2013).

***Longitudinal weights*** The cross-sectional survey weights were already derived by the study teams, while we generated the longitudinal weights. This process began with the creation of a response variable indicating the response and missingness of mental ill-health data in the follow-up waves (ELSA wave 9 and UKHLS-IP wave 13). Propensity scores for this response variable were then calculated using logistic regression, with the response variable as the dependent variable and a set of socio-demographic characteristics at baseline (ELSA wave 6 and UKHLS-IP wave 12) as independent variables. These propensity scores were then merged with the baseline cross-sectional analysis weights to produce follow-up weights that account for response (missingness). The weights were pruned to the 99th percentile and normalised to a sum of 1, yielding the final "longitudinal weights".

**References**

Bhaskaran, K., & Smeeth, L. (2014). What is the difference between missing completely at random and missing at random? *International Journal of Epidemiology*, *43*(4), 1336–1339. https://doi.org/10.1093/ije/dyu080

Firpo, S., Fortin, N. M., & Lemieux, T. (2009). Unconditional Quantile Regressions. *Econometrica*, *77*(3), 953–973. JSTOR. Retrieved from http://www.jstor.org/stable/40263848

Seaman, S. R., & White, I. R. (2013). Review of inverse probability weighting for dealing with missing data. *Statistical Methods in Medical Research*, *22*(3), 278–295. https://doi.org/10.1177/0962280210395740

University Of Essex, I. F. S. (2023). *United Kingdom Household Longitudinal Study; UKHLSUnderstanding Society: Waves 1- , 2008-Understanding Society: Waves 1-13, 2009-2022 and Harmonised BHPS: Waves 1-18, 1991-2009* (Version 18th Edition) [Data set]. UK Data Service. https://doi.org/10.5255/UKDA-SN-6614-18

University of Essex, Institute for Social and Economic Research. (2023). *Understanding Society: Innovation Panel, Waves 1-15, 2008-2022. [Data collection]. 12th Edition. UK Data Service. SN: 6849* [Data set]. https://doi.org/10.5255/UKDA-SN-6849-15.
